# Controlled Li Alloying by Postsynthesis Electrochemical Treatment of Cu_2_ZnSn(S, Se)_4_ Absorbers for Solar Cells

**DOI:** 10.1021/acsaem.3c02483

**Published:** 2023-12-13

**Authors:** Simon Moser, Abdessalem Aribia, Romain Scaffidi, Evgeniia Gilshtein, Guy Brammertz, Bart Vermang, Ayodhya N. Tiwari, Romain Carron

**Affiliations:** † Laboratory for Thin Films and Photovoltaics, Empa—Swiss Federal Laboratories for Materials Science and Technology, Überlandstrasse 129, 8600 Dübendorf, Switzerland; ‡ IMO, Hasselt University, Wetenschapspark 1, 3590 Diepenbeek, Belgium; § IMOMEC, imec, Wetenschapspark 1, 3590 Diepenbeek, Belgium; ∥ EnergyVille 2, Thor Park 8320, 3600 Genk, Belgium; ⊥ ICTEAM, UCLouvain, Place du Levant 3/L5.03.02, 1348 Louvain-la-Neuve, Belgium

**Keywords:** thin-film solar cells, kesterite, CZTSSe, doping and alloying, lithium

## Abstract

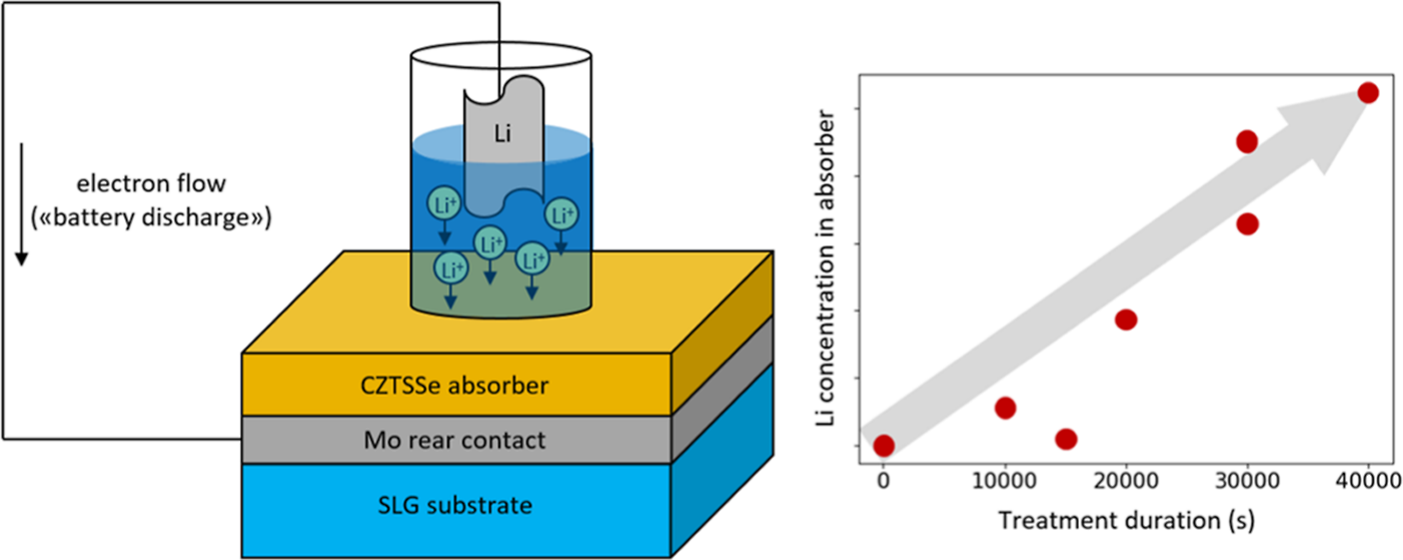

Li-alloying of Cu_2_ZnSn(S, Se)_4_ (CZTSSe) absorbers is widely accepted for its beneficial influence on the performance of CZTSSe-based thin film solar cells. Given the degraded morphology characteristic of absorbers synthesized in the presence of excess Li concentrations, it is speculated that Li may be best incorporated into the absorber after synthesis. Here, we report an innovative method to add Li to synthesized CZTSSe by an electrochemical treatment using a liquid electrolyte. Our approach decouples Li addition from absorber synthesis, allowing one to possibly overcome morphology issues associated with high Li concentration. We show that Li is thereby transferred to the absorber and is incorporated into the crystal lattice. The resulting Li concentration in the absorber can be easily controlled by the treatment parameters. Using liquid electrolytes allows a straightforward disassembly of the lithiation setup and hence the fabrication of solar cells after electrochemical treatment. Electrochemically lithiated solar cells reached power conversion efficiencies of up to 9.0%. Further optimization of this innovative method is required to reduce expected interface issues resulting from the electrochemical treatment to demonstrate a gain in the power conversion efficiency of the CZTSSe solar cells. Finally, our results indicate strong lateral Li diffusion, which deserves further investigation. Moreover, the method could be transferred to other material systems, such as Cu(In, Ga)Se_2_ (CIGS), and adapted to treat layers with other alkali elements such as Na.

## 1 Introduction

Cu_2_ZnSn(S, Se)_4_ (CZTSSe) is a light-absorber material for thin film solar cells, consisting of earth-abundant and nontoxic elements, also referred to as kesterite due to its crystal structure. However, low defect formation energy and narrow phase stability region of CZTSSe increase the open-circuit voltage (*V*_OC_) deficit and thus hampers the power conversion efficiency.^1,2^

Li-alloying is a widely accepted strategy to improve the PV performance of CZTSSe solar cells. The incorporation of Li into the CZTSSe lattice via occupation of Cu-sites increases the band gap of the absorber and widens the unit cell.^3−5^ Moreover, it improves the absorber morphology and increases the apparent carrier concentration.^4,6,7^ Despite various hypotheses—such as inversion of the electric field at the grain boundaries or formation of Li_*x*_Se phases acting as fluxing agents—the mechanism responsible for device performance improvement upon Li-alloying in CZTSSe remains unclear.^6,8,9^ Cabas-Vidani et al. demonstrated 11.6% efficiency with a (Li_*x*_Cu_1–*x*_)_2_ZnSn(S, Se)_4_ absorber grown with a solution-based deposition method.^4^ Although the kesterite crystal structure is maintained upon Li-alloying up to *x* = 0.4,^3^ the highest efficiency was reached at relatively low Li concentrations (*x* = 0.07), with higher Li concentrations leading to degraded morphology and formation of dendritic-shaped features in the absorber. At high Li concentrations, the resulting morphology deterioration neutralizes the beneficial optoelectronic effects of Li-alloying. Consequently, the actual potential of Li-alloying in CZTSSe-based solar cells is still unknown and will only be fully understood if the addition of Li can be decoupled from the absorber synthesis.

Lithium—and more generally alkali elements—can be incorporated into chalcogenide materials at different stages in the absorber fabrication process.^10^ Predeposition processes include the use of an alkali fluoride precursor layer or an alkali-containing Mo back contact. Diffusion from the soda lime glass substrate is a major and often necessary supply source.^10,11^ Alkali elements can also be incorporated during absorber deposition (e.g., coevaporation), or within the precursor layer in the case of solution-based absorber synthesis.^6,11^ Postdeposition, or postsynthesis, treatments (PDT) are the third strategy for alkali incorporation, implemented by, e.g., deposition of an alkali fluoride layer, soaking the absorber in an alkali-containing solution or using so-called CdS-doping.^12,13^ Diffusion of the alkali elements into the absorber is then typically activated by elevated temperatures.^11^

The use of electrochemical methods in the field of chalcogenides has been introduced previously. Electroless deposition has been considered for industrial upscale of thin film solar cell fabrication.^14^ There are two different routes for electroless deposition: (i) the conducting substrate and an easily oxidizable redox component are short-cut and immersed into an electrolyte bath, or (ii) the substrate is immersed into an electrolyte containing dissolved metal salts while using a base metal as counter electrode. The latter procedure even allows one to deposit a precursor stack of multiple elements, using several electrochemical partial reactions simultaneously. The precursor stack can later be transformed into the absorber layer.^15−19^ Electroplating is a very similar technique that requires applied voltage to reduce the ions in the solution and subsequently deposit on the substrate acting as the cathode.^14^ Both methods are only applicable for layer deposition but cannot be used for incorporation into an already existing layer.

We envision high-quality CZTSSe absorbers with high lithium content that do not suffer from deteriorated morphology. To do so, we developed an electrochemical treatment, inspired by the setup of lithium metal batteries, to incorporate Li at ambient temperature into a fully crystallized absorber. Starting with initially Li-free absorbers, we first demonstrate the transfer of Li atoms and homogeneous incorporation into the CZTSSe crystal lattice by this method. Then, the electrochemical treatment is applied on initially Li-alloyed CZTSSe absorbers with good morphology and good PV performance, and the effects of electrochemical Li incorporation on the layer properties and cell PV performance are discussed. Our method allows us to decouple absorber synthesis from Li incorporation, as Li is integrated into the absorber after completion of the growth process.

## 2 Methods

The precursor solution consisted of 0.56 M copper dichloride dihydrate (CuCl_2_·2H_2_O, ≥99.95%, Merck), 0.50 M tin chloride dihydrate (SnCl_2_·2H_2_O, 98.0–103.0%, Thermo Fisher Scientific), 0.44 M zinc chloride (ZnCl anhydrous, 99.95%, Thermo Fisher Scientific) and 1.847 M thiourea (CH_4_N_2_S, ≥99.0%, Merck) dissolved in dimethyl sulfoxide (DMSO, ≥99.9%, Merck). 200–300 nm SiO_*x*_ was sputtered on 5 × 5 cm^2^ soda lime glass (SLG) substrates followed by ∼800 nm Mo. Spin coating was used to deposit the precursor solution on the Mo layer, followed by the subsequent evaporation of the solvent at 320 °C in air. An approximate layer thickness of ∼1.5 μm is reached by repeating the spin coating procedure 10 times. The substrate was cut into 4 quarters, and each quarter was separately annealed using a rapid thermal processing furnace (RTP Annealsys AS ONE 150) inside a semitight graphite box with ∼800 mg of selenium shots (Se amorphous, 99.999+%, Thermo Fisher Scientific). Two temperature plateaus of 350 and 540 °C were held successively for 15 min each. Heating rates were set at 1 K/min, and the annealing environment was N_2_ with a pressure of 500 mbar.

**Figure 1:**
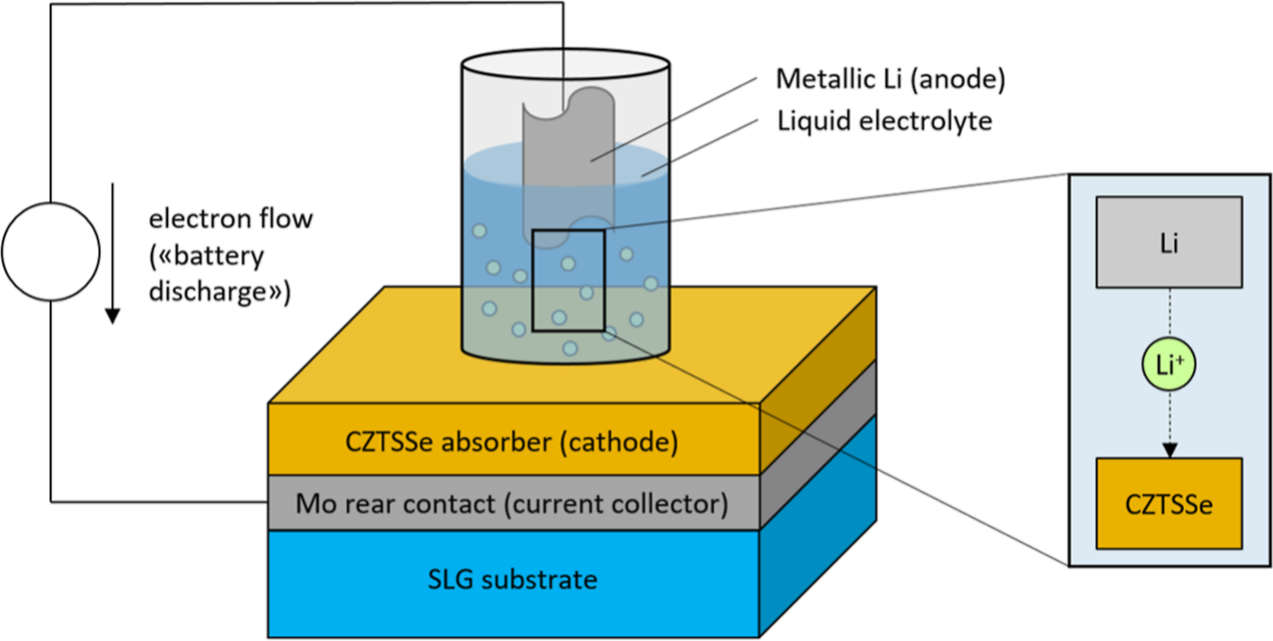
Li incorporation method is shown schematically.

Electrochemical Li incorporation was performed on selenized absorbers in a two-electrode electrochemical cell with a liquid electrolyte, as shown schematically in Figure 1. The cell was connected to a Squidstat potentiostat (Admiral Instruments) inside an argon-filled glovebox (Inert Corp.) at 30 °C. Lithium foil (Merck) was used as the counter electrode. The electrolyte was propylene carbonate with 1.0 M LiClO_4_ (Merck). The cell setup was clamped on the cathode by using a Viton-O-ring for sealing the aperture, thereby defining the active area of the half-cell (0.9 cm^2^). Given the slow decay in voltage upon electrochemical treatment of CZTSSe,^20^ controlling the state of lithiation via the voltage proved challenging. The CZTSSe absorber was instead lithiated by applying a constant discharge current of 5 μA for a set amount of time. The duration of the treatment was used to reach different Li concentrations in the absorber. After the lithiation step, the half-cell was disassembled and the absorber was stored in the glovebox until further use.

To fabricate complete solar cell devices, we applied electrochemical treatment to initially Li-alloyed absorbers. The procedure is identical, but the precursor solution additionally contained 0.253 M lithium chloride (LiCl, 99%, Merck). After electrochemical treatment, the setup was disassembled and the absorbers were rinsed with H_2_O to get rid of the remaining electrolyte on the surface. The absorbers were then immediately immersed into 10 wt % aqueous potassium cyanide (KCN, 97+%, Thermo Fisher Scientific) solution for 30 s to clean the surface from Cu-rich secondary phases. Then, ∼50 nm of the CdS buffer layer was deposited using chemical bath deposition followed by sputtering of 70 and 250 nm of i-ZnO and Al/ZnO, respectively. E-beam evaporation was used to deposit the top grid consisting of 50 nm of Ni and 4000 nm of Al. Finally, each sample was manually scribed into 9 cells with an approximate area of 0.30 cm^2^ each.

Scanning electron microscopy (SEM) images were recorded on a Hitachi S-4800 electron microscope. Time-of-flight secondary ion mass spectrometry (ToF-SIMS) depth profiles were measured on a system from ION-TOF using O^2+^ primary ions with 2 keV ion energy, a current of ∼650 nA and a sputter crater size of 300 × 300 μm^2^. Bi^+^ ions with 25 keV ion energy were used to analyze an area of 100 × 100 μm^2^. *J*–*V* characterization was performed under standard test conditions (100 mW cm^–2^, 22 °C, AM1.5G solar spectrum) using a solar simulator calibrated with a certified Si diode. External quantum efficiency (EQE) spectra were recorded using a chopped white light source (900 W halogen lamp) with a LOT MSH-300 monochromator, and the setup was calibrated with certified Si and Ge diodes. From the resulting EQE spectra, the band gap was determined by using the derivative method. Capacitance–voltage–frequency (CVf) measurements were conducted on the Agilent E4980A Precision LCR meter using a four-probe configuration and 50 mV AC amplitude, with bias and frequency ranging from −1 to 1 V with a 100 mV linear step and from 1 kHz to 1 MHz in a 10-point-per-decade logarithmic sweep, respectively. The samples are brought to low temperature using liquid N_2_ in a semiclosed thermally insulating box while resting on a metal plate connected to a thermocouple and a temperature sensor. X-ray diffraction (XRD) patterns were recorded with 2θ/θ scans using a Bruker D8 diffractometer with Cu Kα radiation (λ = 1.5418 Å, beam voltage = 40 kV, beam current = 40 mA), a step size of 0.05°, a scan rate of 0.5 s/step, and an incident beam size of 2 mm. For higher resolution of the 400 and 008 peaks, a step size of 0.005° and a scan rate of 2 s/step were used instead.

The peak positions of the 400 and 008 peaks were determined by peak deconvolution using two Gaussian functions. Bragg’s law was then used to determine the interplanar distance *d* using the determined peak position, θ, of the respective reflex
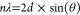
1where *n* is the diffraction order and λ is the wavelength of the incident radiation. Here, the tetragonal crystal system applies so that the lattice parameters *a* and *c* can be derived via
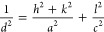
2where *h*, *k*, *l* are the Laue indices of the corresponding crystal plane.

X-ray photoelectron spectroscopy was performed using PHI Quantum 2000 XPS system with a monochromatic Al K_α_ source (1486.6 eV) and a base pressure below 8 × 10^–9^ mbar. The high-resolution scans of the Cu 2p, Zn 2p, Sn 3d, and Li 1s/Se 3d spectra were acquired with a step size of 0.125 eV and a pass energy of 29.30 eV. A charge neutralizer was used for the charge compensation. Ar^+^ sputtering cycles were performed with 2 keV energy. Photoluminescence (PL) spectra were obtained by using a 639 nm diode laser in continuous wave mode and a detection unit from PicoQuant. Raman spectroscopy was performed with a 532 nm laser using an objective magnification of 100×.

## 3 Results

Electrochemical lithiation was performed on bare absorbers for various times: 10 000, 15 000, 20 000, 30 000, and 40 000 s. The samples will be henceforth referred to as 10ks, 15ks, 20ks, 30ks, and 40ks, respectively. 0ks stands for a reference sample, which neither experienced any electrochemical treatment nor was exposed to the electrolyte. The treatment times were chosen to aim at significant Li concentrations of up to 10% Li/(Li + Cu) (see calculation in the Supporting Information). 15ks was part of the sample series but did show delamination of the absorber layer after treatment: therefore, the corresponding experimental results are mentioned in the manuscript but are not part of the analysis.

### 3.1 Lithium Incorporation

**Figure 2:**
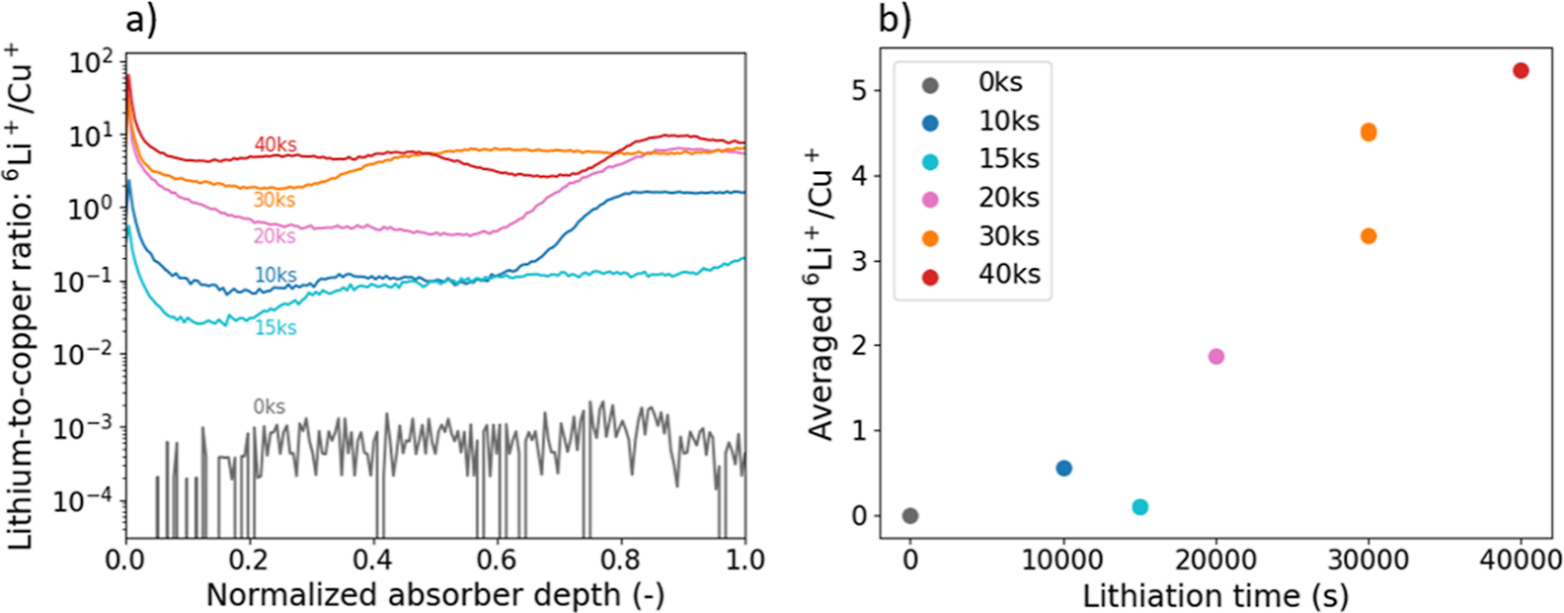
(a) ToF-SIMS depth profiles are shown vs the normalized depth of the absorber. Each lithium signal was normalized with the corresponding copper signal. (b) The depth profile curves were averaged over the whole absorber depth and are plotted against the applied lithiation treatment. Additional data points were calculated for 30ks from two (unshown) curves to indicate the standard deviation.

ToF-SIMS depth profiles were recorded on bare absorbers to verify the incorporation of Li upon electrochemical treatment. Figure 2a shows Li profiles with respect to the normalized absorber depth. The curves are normalized by the intensity of Cu, with Cu being considered constant in all samples due to its low vapor pressure. This assumption has been verified by XRF measurements before and after electrochemical treatment, as the at% values of Cu vary by less than 1%, which is already below the measurement limit of the machine. Furthermore, Cu shows a uniform profile throughout the absorber (Supporting Information, Figure S1). The normalization to Cu allows a reliable comparison between the different curves. The ^6^Li^+^ isotope is used instead of the more abundant ^7^Li^+^, due to detector saturation for the latter. The depth profile corresponding to 0ks shows negligible amounts of Li, as expected. The other curves are all found with higher ^6^Li^+^/Cu^+^ ratios, with most curves showing a homogeneous distribution of Li throughout the absorber. The depth profiles are then averaged over the entire absorber depth and plotted with respect to different lithiation times in Figure 2b. The ^6^Li^+^/Cu^+^ average value increases as the lithiation time increases. 15ks is the only sample that does not follow this trend. For 30ks, various depth profiles were recorded at different spots on the lithiated area, and while in Figure 2a only one curve is shown, Figure 2b contains all three average values to give an idea of the standard deviation.

These ToF-SIMS results illustrated in Figure 2 prove the successful addition of Li into the absorber upon electrochemical lithiation, as the curve for 0ks is found at a factor ∼100 lower ^6^Li^+^/Cu^+^ than almost all lithiated absorbers. Moreover, instead of a single surface layer, Li is homogeneously located throughout the absorber depth, which clearly distinguishes our method from electroplating and electroless deposition.^14^ Variations in ^6^Li^+^/Cu^+^ as a function of absorber depth can be assigned to different causes. On the one hand, the increased intensity toward the top surface of the absorber (depth = 0) can be explained by surface artifacts and potential residuals from the liquid electrolyte. Formation of a solid electrolyte interface (SEI) is also likely, which could result in an increased Li concentration at the top. On the other hand, increased intensity toward the CZTSSe/Mo interface could be explained by voids and smaller grains, which originate from hindered grain growth and the decomposition reaction between CZTSSe and Mo.^21^ The resulting increased grain boundary density offers highly favorable locations for the alkali elements, here Li. ToF-SIMS depth profiles reaching further into the Mo back contact are shown in the Supporting Information (Figure S1) to rule out extensive Li placement in the back contact. Li thus remains in the CZTSSe absorber layer, despite its strong diffusivity.

Furthermore, the average Li concentration in the absorber strongly depends on the lithiation time (Figure 2b). Since the discharge current was kept constant during the lithiation process, it is unsurprising to see such behavior, as the number of Li atoms must be proportional to the number of electrons and hence the product of current and time. Still, it is remarkable that this relationship is directly translated into the absorber, and it points toward a very reliable alkali incorporation technique. It further makes the electrochemical Li incorporation method easily controllable as the number of Li atoms can be simply adjusted by the current or the lithiation time. 15ks is the only sample which does not follow the trend in Figure 2b. As discussed above, delamination of the absorber layer occurred for this sample, and the data point is hence treated as an outlier.

**Figure 3:**
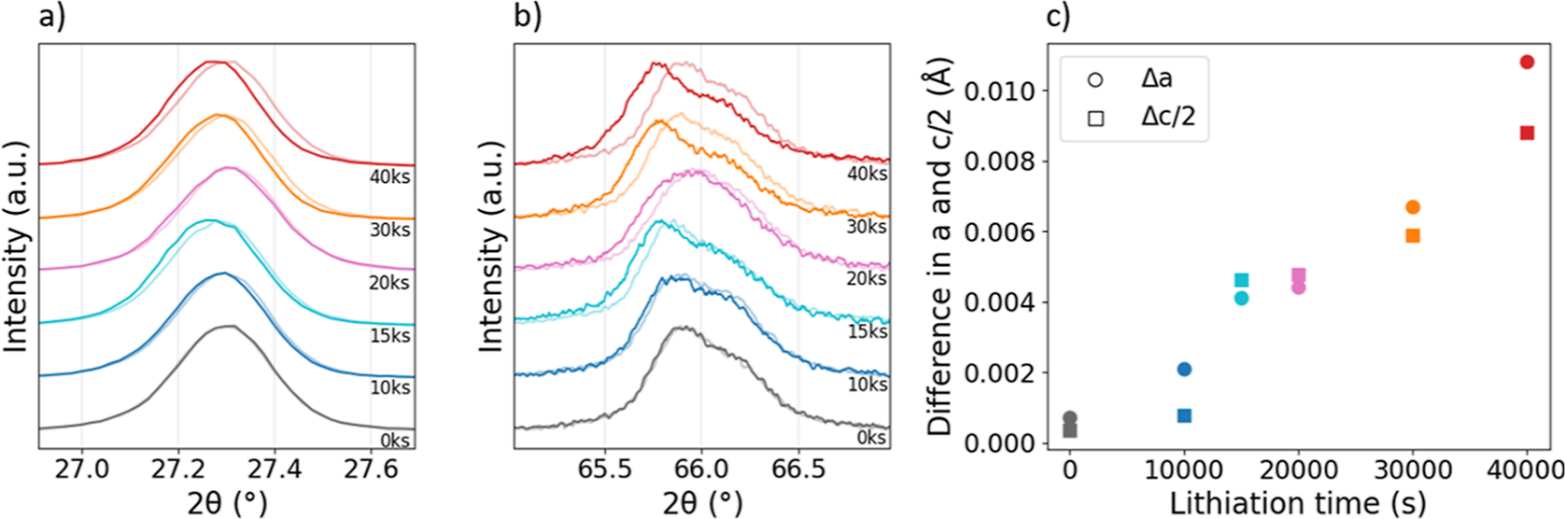
XRD patterns are shown for various lithiation times. (a) The CZTSSe 112 reflex is shown as a zoom-in. Semitransparent lines mark the XRD pattern before the treatment. (b) A zoom-in to the CZTSSe 400 and 008 reflexes is shown. Semi-transparent lines mark the XRD pattern before the treatment. (c) The difference in lattice parameter is calculated from the respective values obtained from peak deconvolution of XRD patterns before and after electrochemical treatment. An increase in lithiation time results in widening of both lattice parameters, *a* and *c*.

To identify the location of Li in the absorber, XRD was performed. Normalized XRD patterns are shown in Figure 3. Figure 3a,b shows close-ups of the 112 and 400 and 008 Bragg reflexes of CZTSSe, respectively. The 400–008 curves were smoothened using a 4 data point rolling average. The semi-transparent lines correspond to diffraction patterns recorded before the electrochemical treatment, which are individually compared to the patterns after the treatment. All peak positions are shifted to lower 2θ angles upon electrochemical treatment. Since the 400 and 008 reflexes partly overlap, a peak deconvolution procedure using 2 Gaussian functions was applied to the raw data to determine the respective peak positions. To avoid spurious fit results, the peak area of the Gaussian function at larger 2θ was imposed to be smaller than the peak area of the Gaussian function at lower 2θ, because the 400 reflex of our absorbers has typically a larger area than the 008 reflex.^4,22,23^ The lattice parameters *a* and *c* were computed according to eqs 1 and 2, and the differences in *a* and *c*/2 before and after treatment are plotted in Figure 3c. It should be noted that lattice parameter *c* is typically twice as large as *a*, which justifies the expression *c*/2. Using a before-after comparison allows one to rule out potential sample-to-sample variations. Both lattice parameters increase with an increasing lithiation time.

The increase in the lattice parameter resulting in unit cell expansion is explained by incorporation of Li into the crystal lattice, thereby ruling out excessive Li accumulation at surfaces, interfaces, voids, or grain boundaries. Occupation of Cu- or Zn-sites by Li is considered the most likely scenario, because the formation energies of Li_Cu_ and Li_Zn_ have significantly lower substitution energy compared to Li_Sn_ and Li_i_, as demonstrated by first-principles calculations.^24^ Our absorbers were fabricated in a Cu-poor and Zn-rich composition with a Cu/Zn ratio of ∼1.45, determined by XRF. Therefore, *V*_Cu_ is more abundant than *V*_Zn,_ so occupation of Cu-sites is considered more likely. The bond valence parameter of Li–Se is larger than for Cu–Se.^25^ Cu-site occupation by Li hence explains the increase in lattice parameter and has been reported by numerous recent studies.^3−5,23^ Cabas-Vidani et al. and Lafond et al. observed a change in lattice parameter *a* only and assigned that to the occupation of Wyckoff 2a sites by Li,^3,4^ while Yang et al. found that Li incorporation into the lattice changes the lattice parameter *c* only.^5^ Here, both lattice parameters *a* and *c*/2 increase very similarly upon Li incorporation, with the cause of this inconsistency remaining unclear.

XRD results show that our method is capable of incorporating Li into the crystal lattice to form Li-alloyed CZTSSe even after the absorber synthesis. The electrochemical process does not require a heat treatment as it is commonly used, e.g., standard alkali fluoride postdeposition treatments.^11^ Additional experiments were conducted to investigate the influence of heat treatment—15 min at 300 °C in an N_2_:Se protection atmosphere—after electrochemical lithiation and can be found in the Supporting Information (Figure S2). No significant difference in the XRD pattern after heat treatment confirms that Li incorporation occurs regardless of the available thermal energy.

Furthermore, it is striking that the increase in the lattice parameter due to Li-alloying is about proportional to the lithiation time. It once again emphasizes the controllability of the electrochemical lithiation technique. It should also be noted that the 15ks sample, which is considered as an outlier as discussed before, again slightly deviates from the observed trend.

Finally, full-range diffraction patterns are shown in the Supporting Information (Figure S3). No secondary phases could be identified upon electrochemical treatment at short lithiation times. The kesterite phase is thus expected to remain intact. Yet, most secondary phases related to CZTSSe coincide with the CZTSSe Bragg reflexes and are thus overlapping.^26^ At longer lithiation times, additional reflexes appear at ∼13.3° and ∼23.0°, indicating formation of parasitic phases.

### 3.2 Lateral Li Diffusion

Surprisingly, Li is found not only in the part of the absorber that was in contact with the electrolyte. Additional ToF-SIMS measurements on 30ks and 40ks show Li in the whole absorber layer. Therefore, it is assumed that Li is capable of diffusing laterally. However, the amount of Li found adjacent to the lithiation spot varied strongly in the investigated absorbers. While the averaged ^6^Li^+^/Cu^+^ ratios for 30ks on and adjacent to the lithiation spot are very similar, the adjacent ^6^Li^+^/Cu^+^ ratio for 40ks is strongly reduced (Supporting Information, Figure S4). The speed of this diffusion hence remains unclear. XRD measurements adjacent to the lithiation spot even indicate that Li could be incorporated into the crystal lattice of the full absorber. However, the available data on lateral Li diffusion is limited to only few measurement spots and few absorbers, thus not allowing reliable conclusions. Lateral Li diffusion deserves extensive investigation, which is beyond the scope of this work.

### 3.3 Quantification of Li Incorporation

**Figure 4:**
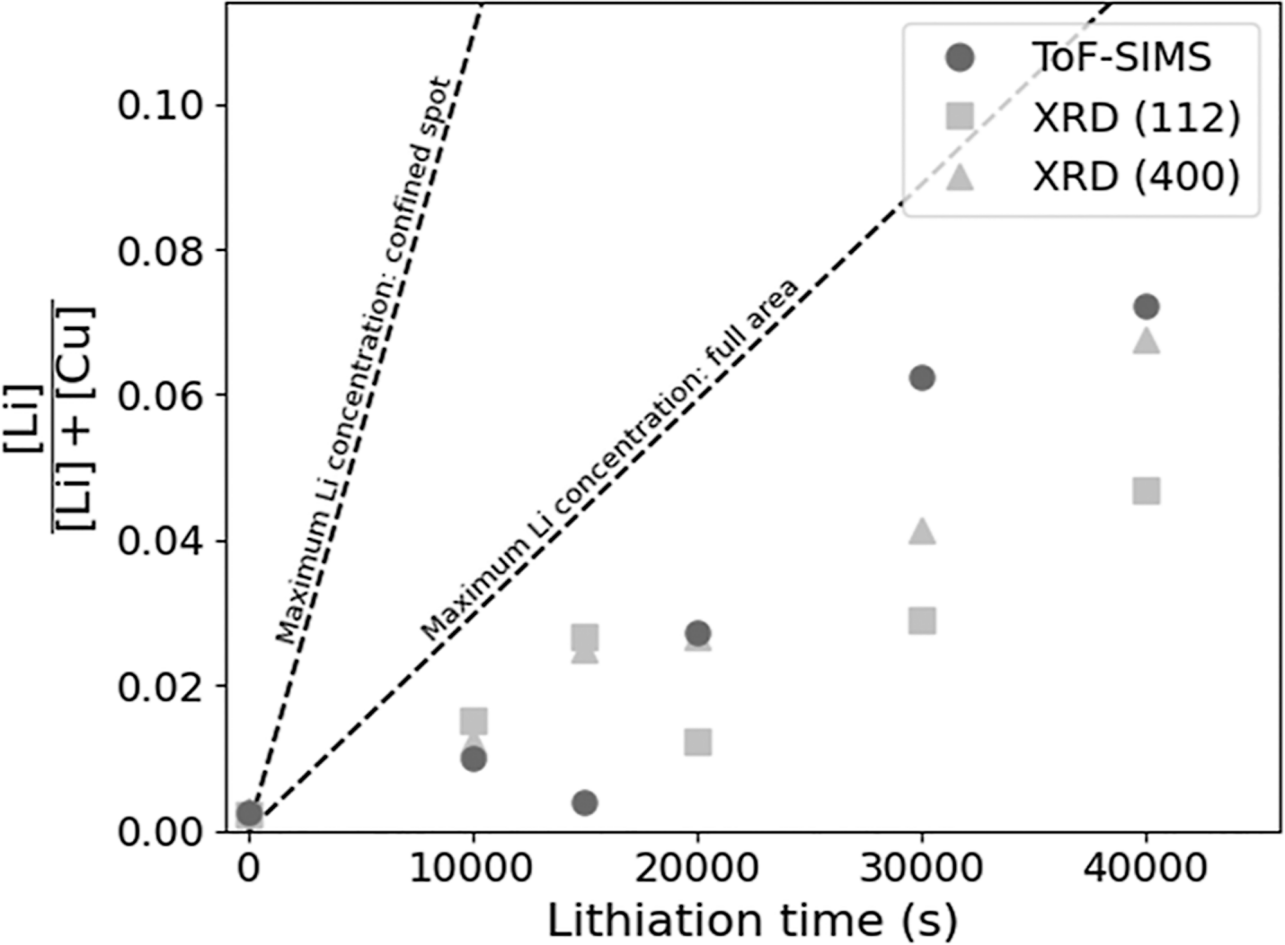
Li/(Li + Cu) ratio is calculated from various methods. The product of lithiation time and current is proportional to the maximally available amount of Li and is shown for different areas (−). The amounts determined from the ToF-SIMS depth profiles (●) mark the amount of Li placed in the absorber. From XRD (▲ and ■), Li occupying the lattice sites can be quantified. Quantification from ToF-SIMS and XRD was realized via calibration with ICP–MS results based on a recent work from Cabas-Vidani et al.^4^

The various characterization methods were used to quantitatively evaluate the Li concentration in the absorber and in the crystal lattice (Figure 4). First, the maximum available Li content in the system was calculated from the discharge current and the respective lithiation time of the electrochemical treatment. The number of transferred Li atoms is equal to the number of transferred electrons. Given the uncertainty of lateral Li diffusion, the resulting concentration is calculated for both the confined lithiation spot area and full absorber area. The detailed calculations can be found in the Supporting Information. Second, the amount of Li is calculated from the ToF-SIMS ^6^Li^+^/Cu^+^ average values, as well as from the 400 and 112 XRD peak shifts. In order to do that, a recent work of our research group from Cabas-Vidani et al. was used as calibration, with details being reported in the Supporting Information.^4^ They reported ToF-SIMS, XRD, and ICP–MS results for Li-alloyed CZTSSe absorbers with various Li amounts obtained on the same characterization equipment. There is good agreement between the curves derived from the 400 and 112 reflex positions. Since Cabas-Vidani et al. observed an increase only in lattice parameter *a*, and lattice parameters *a* and *c* changed in this work, the curve originating from the 112 XRD reflex is used for the following discussion.

The Li concentration for the untreated absorber calculated from ToF-SIMS and XRD is >0 in Figure 4, which is considered an artifact, either in our experimental data or in the data used for calibration. Yet, all curves show an essentially linear increase in Li/(Li + Cu) concentration as a function of lithiation time, which was expected from the previous results. It again emphasizes the high controllability of the electrochemical Li-alloying method. As discussed before, 15ks delaminated after treatment and is hence not considered for interpretation in this section. Comparison of the data points originating from ToF-SIMS with the curves for maximum Li concentration allows us to estimate the amount of lost Li during the process. If Li were only present in the absorber volume below the lithiation spot, the estimated Li loss would be significant, but comparable to the Li loss experienced in solution-based Li-alloying.^4,6^ If Li were capable of diffusing to the full area of the absorber, then the Li loss would be significantly lower. As has been discussed before, there is evidence that Li does diffuse laterally and also occupies sites in the adjacent area of the absorber. Therefore, the actual maximum Li concentration could lie somewhere between both curves. One possible explanation for the loss of Li is that the electrolyte is first saturated with Li before the ions move into the absorber. SEI formation, which has been discussed before, could also contribute to an apparent loss in Li. Another explanation is the incorporation of Li into other layers of the sample, e.g., Mo or the SLG substrate. The latter mechanism is, however, unlikely as the ^6^Li^+^ intensity is drastically reduced when reaching the Mo back contact, as seen from the ToF-SIMS depth profiles shown in the Supporting Information (Figure S1).

Next, the data points originating from ToF-SIMS and XRD are compared. While ToF-SIMS allows to make claims about the quantity of Li found in the absorber, XRD only takes into account Li occupying lattice sites, excluding, e.g., grain boundaries. Up to a lithiation time of 10 000 s, the Li concentrations derived from ToF-SIMS and XRD are similar. We hence believe that most of the Li in the absorber is incorporated into the lattice at short lithiation times. When longer lithiation times are applied, there is a gap between the ToF-SIMS and XRD data points, suggesting that a significant amount of Li may be present in the absorber, which does not occupy Cu sites in the crystal lattice. As discussed before, we believe that Li is favorably placed on *V*_Cu_ sites because of the similar ionic radius of Cu^+^ and Li^+^, the low formation energy of Li_Cu_, and the abundance of *V*_Cu_ in Cu-poor kesterite. With progressing lithiation, more and more of the *V*_Cu_ sites are occupied, so it becomes more and more unlikely to place each incoming Li on a *V*_Cu_ site, and it becomes more likely for Li to be placed elsewhere. Alternative locations could be grain boundaries, voids, or interstitials. Although the Li concentrations originating from the 400 and 112 XRD reflexes are slightly different, the resemblance of the curves supports both the reliability of the data and the significance of the calibration method.

### 3.4 Analysis

XPS was performed on absorbers fabricated in the same way as 0ks and 10ks – referred to as 0ks′ and 10ks′—to investigate the effects of lithiation on the chemical states within the layer. The XPS results can be found in the Supporting Information (Figures S5 and S6). The initial comparison between 0ks′ and 10ks′ was made based on the measurements performed on the surface, as no Ar^+^ presputtering was performed. Only the surface was in contact with the electrolyte during the Li treatment. The surface is thus expected to have the highest probability of showing possible deviations. The absence of any significant difference between the two samples regarding the ratio of the peak intensities means that a potential loss of Cu, Zn, and Sn upon electrochemical lithiation is below the detection limit of XPS (0.1–1.0 at %).^27^ The Sn 3d peak is observed to shift by ∼0.1 eV toward lower binding energies upon lithiation, which could indicate a reduction of Sn. Next, the XPS measurements from the treated absorber 10ks′ were obtained after ion sputtering to more than 500 nm in the bulk of the absorber thickness. The comparison with the surface does not show significant differences either, ruling out bulk effects upon electrochemical treatment. Only the Cu 2p peak shows increased intensity at the surface, which is rather assigned to the absence of KCN etching on the characterized absorbers. KCN etching is commonly used to remove Cu-rich phases—and Sn-rich phases to a lesser extent—at the absorber surface.^28,29^

**Figure 5:**
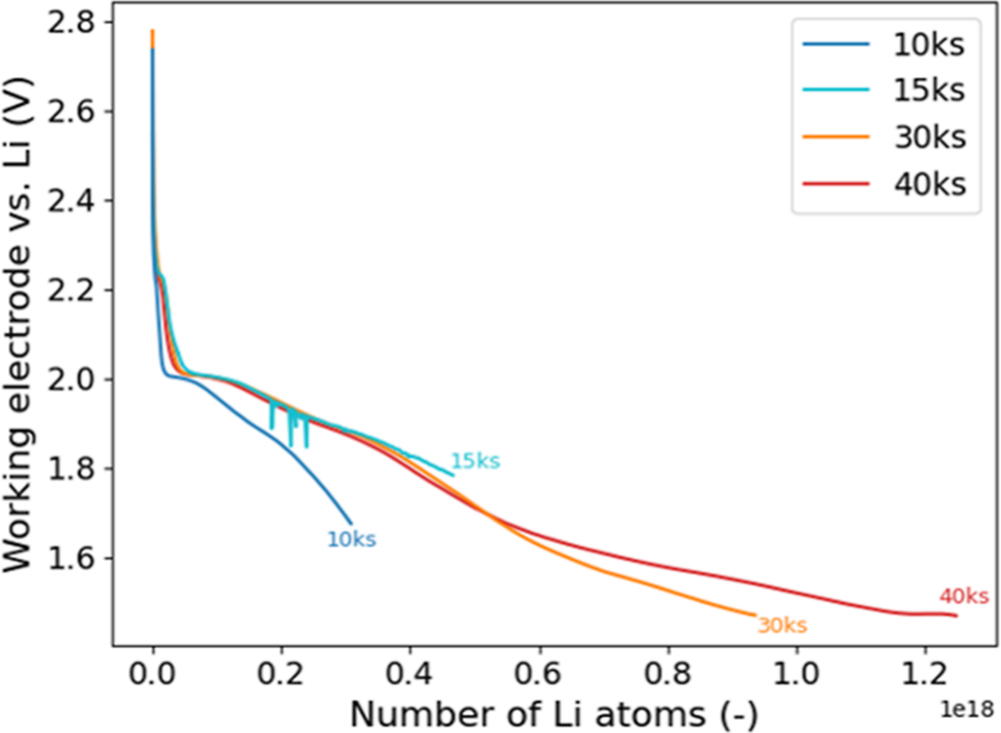
Discharge curves for the electrochemically lithiated absorbers. The *x*-axis shows the cumulative number of Li atoms calculated from the lithiation time and the current.

Figure 5 illustrates the voltage profiles during the lithiation (i.e., discharge) process, revealing five plateaus^30^ at approximately 2.2, 2.0, 1.9, 1.6, and 1.4 V for 15ks, 30ks, and 40ks. These curves are consistent, while 10ks exhibits a more significant decrease in voltage with respect to the number of Li. The shown curve for 15ks consists of the envelope of the data set, to smoothen fluctuations probably occurring due to simultaneous activities in the glovebox affecting the data quality. The curve of 20ks is not shown due to potentiostat calibration issues but can be found in the Supporting Information (Figure S7).

Initially, discharge curves overlap; however, differences emerge starting from approximately 2.0 V. Such differences are common in thin-film electrochemical behavior and are attributed to exposure of deeper regions to the liquid electrolyte due to proceeding lithiation.^31,32^ This effect is particularly pronounced when reactions involve substantial volume expansion, for example, the lithiation of pure Sn, which exhibits a nearly 90% volume increase.^33^ Different amounts of deep material exposed could explain the deviation of the 10ks discharge curve compared to the other curves.

We hypothesize that the region between 2.2 and 2.0 V is associated with Se lithiation.^34^ A possible mechanism according to literature is the reaction with Cu species, including e.g., residual Cu_*x*_Se, which could be present in small amounts in the absorber.^35−37^ The region between 2.0 and 1.4 V could be attributed to Li insertion, partially forming the desired (Li_*x*_Cu_1–*x*_)_2_ZnSn(S, Se)_4_ phase, while a reduction reaction is probably taking place for Cu, Zn, or Sn.^20,38^ It has been reported that Sn is predominantly reduced, which would align well with the observed peak shift in XPS (Figure S5c).^36,37^ Another plateau is reached at 1.4 V, which possibly arises from further conversion of (Li_*x*_Cu_1–*x*_)_2_ZnSn(S, Se)_4_, into Li_2_(S, Se), lithiation of Sn resulting in Li_*x*_Sn alloys, and SEI layer formation, consistent with previous reports on kesterite lithiation.^20,36,38,39^ In contrast to the literature, which is based on pure sulfur kesterite, here, CZTSSe with a S/Se ratio of ∼5% is used, which reflects higher voltage values.^23^

Based on these hypotheses, the discharge curves suggest a reaction involving Se before lithiation of the CZTSSe phase starts. Such a reaction is unwanted, as it could destroy the kesterite phase. But, since XRD (Figures 3 and S3) confirms the presence of (lithiated) kesterite phase without significant reduction in peak intensity after electrochemical treatment, Se lithiation is either only a minor reaction taking place, or Se indeed originates from secondary phases such as Cu_*x*_Se. The lithiation mechanism of CZTSSe takes place between 2.0 and 1.4 V, forming a more Li-rich (Li_*x*_Cu_1–*x*_)_2_ZnSn(S, Se)_4_ phase the longer the electrochemical treatment is maintained. This is equivalent to an increase in Li/(Li + Cu).

It has been extensively discussed before that the majority of Li is located in the crystal lattice, most probably on Cu vacancies (Figures 3 and 4). Li-alloying of CZTSSe usually results in the widening of the band gap according to many previous reports in the literature.^3−5,23^ PL spectra were recorded on 20ks, 30ks, and 40ks before and after electrochemical treatment (Supporting Information, Figure S8). Due to the low PLQY of CZTSSe, high excitation intensities of up to 5 W cm^–2^ (≙50*sun) had to be used, which could explain the differences in PL peak intensity, as the diode ideality factor might change upon treatment. The PL maximum energies were determined and are reported in Table 1. Although a slight shift of the peak position of up to 9 meV is visible in the PL spectra, it is clearly less pronounced than would have been expected. Based on the quantification in Figure 4 and the findings on the band gap from Cabas-Vidani et al., 40ks contains ∼4% of Li, which should reflect in an increase of the band gap and thus a shift in the PL maximum energy by at least 70 meV.^4^

**Table 1:** PL Maximum Energy Reported for 20ks, 30ks, and 40ks before and after Treatment

	before treatment (eV)	after treatment (eV)
20ks	1.023	1.021
30ks	1.017	1.021
40ks	1.023	1.032

As the presence of Li-alloying upon electrochemical lithiation was undoubtedly proven, there must be a second mechanism counteracting the related band gap widening. A possible mechanism is the intercalation of the remaining Li atoms as interstitials. As discussed in Figure 4 comparing Li concentration estimates based on ToF-SIMS and XRD, there is a significant share of Li in the absorber, which does not contribute to XRD peak shifts, and hence is not placed on vacant Cu-sites. Although the formation energy of Li_i_ is significantly higher than that of the occupation of an empty Cu-site, its occurrence cannot be ruled out. The actual effect of Li_i_ can only be hypothesized, as it has never been observed experimentally for CZTSSe, probably because of its unlikeliness. However, several works claim that Na_i_ causes a narrowing of the band gap, due to the first conduction band broadening.^40,41^ The chemical similarity of Li and Na could result in a mechanism similar to that based on Li_i_. Consequently, the simultaneous occurrence of Li_Cu_ and Li_i_ could lead to the observation that the band gap appears to remain unchanged.

A change in the S/Se ratio in CZTSSe could also influence the band gap.^7^ Absorbers used in this work have a S/Se ratio of ∼5%.^23^ Assuming a complete loss of S upon electrochemical treatment for CZTSSe with a S/Se ratio of 5%, the band gap decrease is expected up to 0.025 eV, according to Vegard’s law.^42^ As discussed before, the expected band gap increase resulting from a 4% Li/(Li + Cu) ratio is in the region of 0.070 eV.^4^ Therefore, a change in the S/Se ratio does not have the capability of entirely counteracting the Li-alloying-induced band gap widening by itself.

Sn loss upon electrochemical lithiation is another possible explanation for the absence of a band gap shift. Azzouzi et al. have shown that the band gap in CZTSSe is affected by the amount of Sn and that a reduction of Sn results in the narrowing of the band gap.^43^ As has been discussed before, not only Li incorporation into the crystal lattice but also several conversion reactions are hypothesized to take place upon electrochemical treatment. Those, which involve the reduction of Sn, could lead to a decreased Sn concentration of the CZTSSe phase. We believe that Sn-related conversion mechanisms are especially relevant for long lithiation times due to the corresponding voltage levels, as discussed before (Figure 5). Such a mechanism, or a similar conversion reaction, could possibly explain the appearance of so far unidentified reflexes at ∼13.3° and ∼23.0° for long lithiation times visible in full-range XRD patterns (Figure S3). In general, variations of the chemical composition of the cations, which are below the detection limit of XPS, could be present (Supporting Information, Figure S5). Therefore, Sn loss and other chemical fluctuations cannot be ruled out and could thus be responsible for the missing band gap shift upon electrochemical lithiation.

Cu/Zn disorder is another factor affecting the band gap in CZTSSe,^3,44^ and would alter the XRD peak position as well.^45^ Raman spectroscopy (Supporting Information, Figure S9) was performed on an electrochemically treated absorber (40ks). Scragg et al. assigned general peak broadening and changes in relative intensity of the main peaks to Cu/Zn disorder.^46^ Here, no comparable behavior was observed, so a change in Cu/Zn disorder is considered negligible and hence is most likely not reflected in the band gap.

### 3.5 Solar Cells

**Figure 6:**
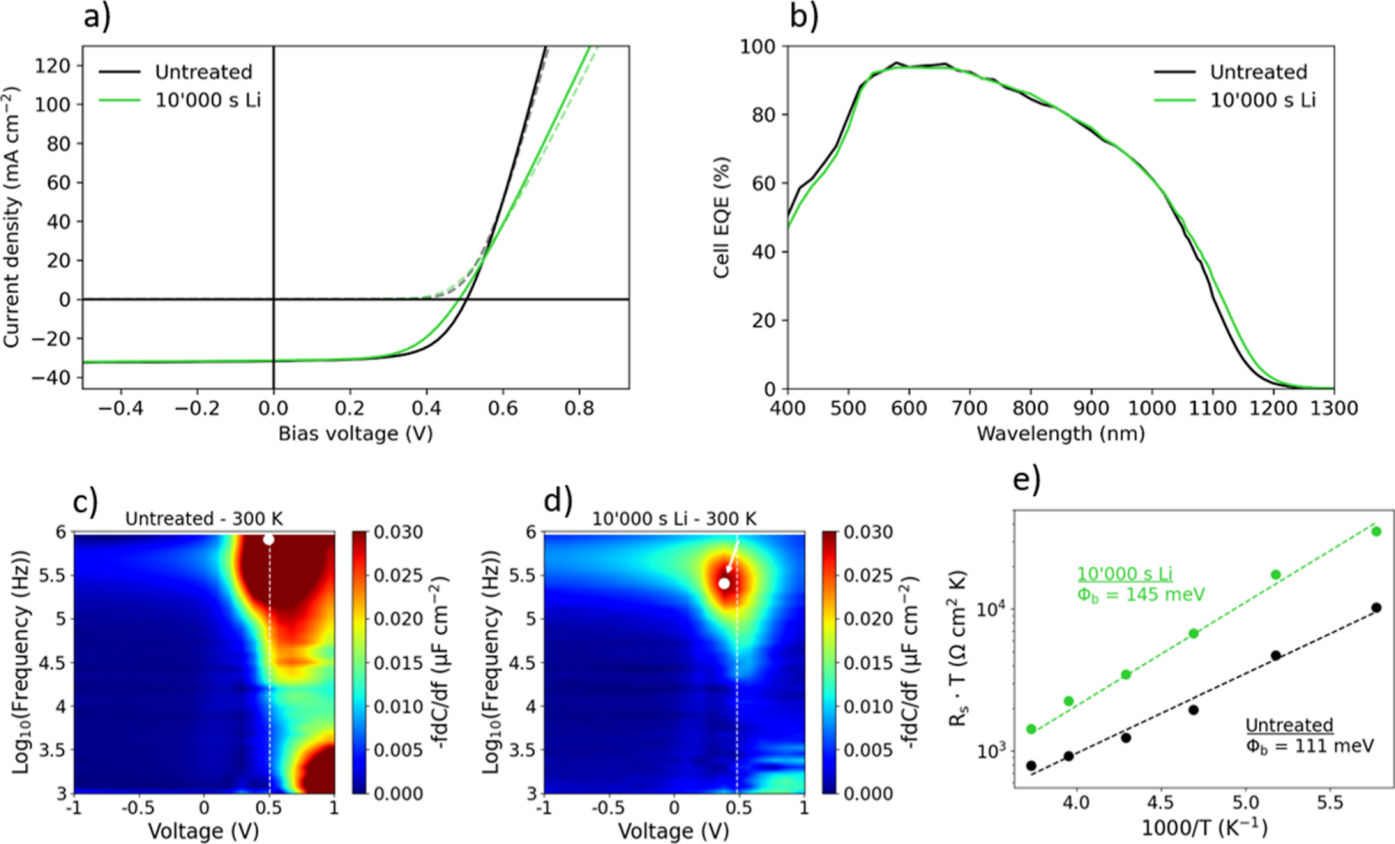
Devices were fabricated from initially Li-alloyed CZTSSe absorbers. (a) Illuminated *J*–*V* curve from an untreated reference device (black) and a nominally identical cell with additional electrochemical treatment (green). (b) Corresponding EQE spectra of the cells shown in subfigure (a). (c,d) Bias-dependent admittance spectroscopy CVf maps for the untreated and additionally electrochemically lithiated devices, respectively. The white dashed line represents the value of *V*_OC_ for each device, while the white dots and white arrow, respectively, highlight the interface response peak and its modification after lithiation. (e) Corresponding Arrhenius plots of *R*_s_·*T* vs 1000/*T* for the untreated (black dashed line and symbols) and additionally electrochemically lithiated device (green dashed line and symbols), obtained via semicircle fitting of the Nyquist plots coming from low-temperature admittance spectroscopy measurements.

PV devices were fabricated to prove the compatibility and potential of electrochemical lithiation for CZTSSe absorber fabrication. Illuminated *J*–*V* curves, EQE spectra, and bias- and temperature-dependent admittance spectroscopy are shown in Figure 6. It is understood that grain growth during CZTSSe absorber fabrication heavily depends on the presence of fluxing agents such as Li_2_Se.^23^ Therefore, initially Li-containing absorbers (Li was added via the precursor solution) with high power conversion efficiency levels were chosen for this section instead of Li-free absorbers. Electrochemical lithiation was used as an additional Li incorporation method after synthesis. By that, achieving higher Li concentration than declared as optimum by previous reports was anticipated, while still maintaining a favorable morphology of the absorber.^4^ As a proof of concept, a relatively mild treatment of 10 000 s was applied. Figure 6a and Table 2 show the *J*–*V* curves and the device properties of the treated absorber device and the untreated reference device. Table 2 additionally shows the standard deviation of the untreated device determined on a total of 9 cells in the same sample. Moreover, the treatment of the lithiated device was repeated on another device, and the resulting PV properties are reported in the Supporting Information (Table S1). While the lithiation does not significantly affect the short-circuit current (*J*_SC_), *V*_OC_ and the fill factor are slightly degraded. The EQE spectra depicted in Figure 6b do not show significant differences among the samples. The respective values for the band gaps were derived from the EQE spectra and are reported in Table 2. For bias-dependent admittance spectroscopy CVf maps, so-called “loss maps,”^47^ shown in Figure 6c,d, the most noticeable difference is the top right corner. The response is slightly shifted toward lower bias voltage and lower frequency for the lithiated absorber compared to the untreated absorber (white arrow in Figure 6d). The exponential evolution of series resistance (*R*_s_) with temperature on the Arrhenius diagram in Figure 6e reveals a higher activation energy—or barrier height φ_b_—for the lithiated device.

**Table 2:** Summary of the PV Properties of Devices Based on an Untreated and an Electrochemically Lithiated Absorber^a^

	*V*_OC_ (mV)	*J*_SC_ (mA cm^–^^2^)	FF (%)	PCE (%)	*R*_s,ill._ (Ω cm^2^)	*R*_p,dark_ (Ω cm^2^)	*E*_g_ (eV)
untreated	505 ± 11	31.7 ± 1.3	62.4 ± 5.0	10.0 ± 0.8	0.5 ± 0.3	11 820 ± 9890	1.130
10 000 s Li	485	31.4	58.8	9.0	1.7	16 780	1.125

a Both absorbers were initially Li-alloyed. The ± value shows the 2σ standard deviation determined on the untreated sample over 9 individual cells. The sample-to-sample variation could be different from this value.

We then verified that the electrochemical treatment has the same effects on initially Li-alloyed absorbers as on initially Li-free absorbers. XRD patterns were recorded before and after the treatment (Supporting Information, Figure S10), and the resulting lattice parameter changes were 0.0019 and 0.0015 Å for *a* and *c*, respectively. These values align well with the lattice parameter shifts for sample 10ks, found at 0.0021 and 0.0015 Å for *a* and *c*, respectively (Figure 3c), proving the applicability of electrochemical lithiation on already Li-alloyed absorbers. Thus, the results on initially Li-free absorbers can directly be applied to initially Li-alloyed CZTSSe absorbers. Strikingly, the absorber morphology is not significantly affected by the electrochemical treatment of an initially Li-alloyed absorber (Figure S11). This observation is assigned to the capability of our method to incorporate Li at room temperature without the need for thermal energy, thus preventing atoms from becoming mobile again. Therefore, electrochemical lithiation is a possible strategy to overcome morphology issues in Li-alloyed CZTSSe absorbers.

The decreased performance level of the lithiated device can be attributed to the lowered *V*_OC_ and fill factor. However, the reduction of *V*_OC_ by only 20 mV is within the expected sample-to-sample variation. Figure 6b shows the EQE curves for the untreated device and the lithiated device, which rules out a band gap effect as the origin of the *V*_OC_ difference, and the unchanged band gap aligns well with the previously discussed PL data (Table 1). Moreover, the similarity between the EQE curves rules out collection problems of the lithiated cell. Therefore, the main consideration for the slightly worse PV performance of the electrochemically lithiated device is the degraded fill factor, which is a result of the increased series resistance (Table 2). The significance of the increase in series resistance is confirmed by the data on the repeated cell reported in the Supporting Information (Table S1), and the standard deviation reported in Table 2. Other parameters possibly affecting the fill factor can be ruled out to be the cause of the degradation. As visible from Table 2, the shunt resistance (*R*_p_) remains at a benign level and the electrical diode ideality factor obtained from *J*_SC_–*V*_OC_ measurement does not deteriorate drastically on the lithiated cell (1.19 vs 1.22), as shown in the Supporting Information (Figure S12).

A possible explanation for *R*_s_ degradation could be due to interface modifications as a result of the contact between the absorber and electrolyte during electrochemical treatment. A reaction between kesterite and liquid electrolyte has been previously reported for kesterite anode materials.^20^ Admittance spectroscopy was used to study the interface modifications of the devices upon lithiation. Representing the evolution of the capacitance derivative with respect to frequency (−*f*d*C*/d*f*) as a function of both voltage and frequency on a 2D plot enables identification of potential loss mechanisms and their physical nature, as studied in a recent work by Brammertz et al.^47^Figure 6c,d show such 2D plots for a treated and a reference cell. The bottom right corner response can be ignored since it is a consequence of large forward currents impeding accurate capacitance measurements.^47^ Thus, the most exciting feature observed on both experimental maps is one prominent peak located around *V*_OC_ and beyond 100 kHz with a tail extending toward negative voltages. Comparing the experimental “loss maps” shown here in Figure 6 with simulated “loss maps,”^47^ the feature could either be associated with a bulk defect in the absorber, or with either a spike-like barrier or a defect in the absorber/buffer interface, or even with a combination of both. However, the 100 meV discrepancy between extrapolated *V*_OC_ at 0 K and the absorber bandgap shown in the Supporting Information (Figure S13) suggests that the *V*_OC_ is not bulk-limited on the first order, which supports the pn-junction interface as the main source of losses via a barrier or a defect.

To distinguish between possible interface mechanisms, admittance spectroscopy measurements are performed at low temperatures. In the corresponding Arrhenius plot, shown in the Supporting Information (Figure S14), the energy level of the defect around 100 meV away from the neighboring band edge tends to correlate well with the 100 meV discrepancy between extrapolated *V*_OC_ at 0 K and the absorber band gap, shown in the Supporting Information (Figure S13). The interface defect thus maintains its potential importance, concerning the high *V*_OC_ deficit of both cells, which remains common for CZTSSe. Still, there is no significant change in either the energy level or the capture cross section between the untreated and lithiated device. Therefore, electrochemical lithiation probably does not modify the energy level of the interface defect. On the other hand, corresponding Nyquist plots are fitted by semicircles, and in the case of a potential barrier at the absorber/buffer interface, the associated series resistance can be extracted as the left-side intersection with the horizontal axis of the fit for each temperature.^48^ The data are fitted using the exponential law for thermionic emission, which unveils a potential barrier higher by around 35 meV for the electrochemically lithiated device (Figure 6e), resulting in a larger associated resistance. This barrier could possibly explain *V*_OC_ and fill factor degradation upon lithiation.

Finally, the *J*–*V* curve and the CVf map for a cell adjacent to the lithiation spot but on the same absorber are shown in the Supporting Information (Figure S15). Assuming unconstrained lateral Li diffusion, the absence of *R*_s_ degradation on the adjacent cell strongly suggests that contact with the electrolyte is responsible for the deteriorated PV performance of the lithiated cell. Yet, further work is required to unambiguously identify lateral Li diffusion to verify this assumption. Even more so, since lateral Li diffusion could be the key to overcome the current limitations of electrochemical lithiation. That is, if the lateral Li diffusion mechanism can be confirmed, the treatment could be applied at an irrelevant area on the absorber, which would thereby be sacrificed in order to lithiate the rest of the absorber.

## 4 Conclusions

In summary, we demonstrate a new electrochemical treatment method to incorporate Li into CZTSSe after absorber synthesis. Quantification of Li based on ToF-SIMS and XRD hints that the majority of Li is incorporated into the crystal lattice as opposed to, e.g., plating, segregation, or accumulation at grain boundaries. Li-alloying of CZTSSe is thereby decoupled from absorber synthesis, allowing one to benefit from favorable morphology in high-Li absorbers, which has not been possible with conventional strategies so far. Furthermore, we found evidence of strong lateral Li diffusion, as Li has been found in the whole absorber layer and not only on the confined lithiation spot. Additional in-depth analysis is required to confirm and better understand this ion migration.

Disassembly of the lithiation setup is straightforward after electrochemical treatment thanks to the use of a liquid instead of a solid electrolyte, enabling the subsequent completion of solar cell devices. We achieved a remarkable power conversion efficiency of 9.0% without antireflective coating with treated absorbers, with slight losses compared to an untreated reference mainly coming from deteriorated *R*_s_. Electrical analysis suggests that electrochemical lithiation influences the barrier height at the CZTSSe/CdS interface, which could explain the degraded fill factor due to more prominent *R*_s_ and slightly deteriorated *V*_OC_.

Although only a mild treatment was applied before PV device fabrication, it is a highly promising result and could pave the way toward high-quality and high-Li CZTSSe absorbers, with the potential to reach higher power conversion efficiencies in the future. Further work is required to optimize the treatment to circumvent this *R*_s_ degradation limiting device performance. Beyond Li in CZTSSe, our method could also be applied to other alkali elements such as Na and to other material systems such as CIGS.

## Data Availability

Data displayed in the graphs of the manuscript is available at https://zenodo.org/communities/custom-art/?page=1&size=20 under DOI 10.5281/zenodo.8134310 (will be published on Zenodo as soon as the manuscript is accepted).

## Supporting Information Available

Calculated number of incorporated Li ions; ToF-SIMS depth profiles for Cu^+^ and ^6^Li^+^; XRD before and after post-treatment annealing; full-range XRD patterns before and after treatment; ToF-SIMS and XRD analysis of spot adjacent to lithiation treatment; details of calculation for Li quantification; XPS spectra; discharge curves; PL spectra; Raman; additional JV data; additional characterization of the device (discharge curve, XRD); SEM cross-section; *V*_OC_–*J*_SC_; *T*-dependent *V*_OC_; Arrhenius plot *C*–*f* measurement; and additional characterization of the device adjacent to lithiation treatment (PDF)
