# Supplementary material for: Controlled Li Alloying by Postsynthesis Electrochemical Treatment of Cu2ZnSn(S, Se)4 Absorbers for Solar Cells
Source: ACS Appl Energy Mater. 2023 Dec 13;6(24):12515–25. doi: 10.1021/acsaem.3c02483 (PMC10751737; doi:10.1021/acsaem.3c02483)
Supplement: Supplementary file 1 — ae3c02483_si_001.pdf [file ae3c02483_si_001.pdf]

Supporting Information

Controlled Li Alloying by Post-Synthesis Electrochemical Treatment of $Cu_2ZnSn(S, Se)_4$ Absorbers for Solar Cells

Simon Moser[a,*], Abdessalem Aribia[a], Romain Scaffidi[b,c,d,e], Evgeniia Gilshtein[a], Guy Brammertz[b,c,d], Bart Vermang[b,c,d], Ayodhya N. Tiwari[a], Romain Carron[a]

[a] Laboratory for Thin Films and Photovoltaics, Empa – Swiss Federal Laboratories for Materials Science and Technology, Überlandstrasse 129, 8600 Dübendorf, Switzerland

[b] IMO, Hasselt University, Wetenschapspark 1, 3590 Diepenbeek, Belgium

[c] IMOMEC, imec, Wetenschapspark 1, 3590 Diepenbeek, Belgium

[d] EnergyVille 2, Thor Park 8320, 3600 Genk, Belgium

[e] ICTEAM, UCLouvain, Place du Levant 3/L5.03.02, 1348 Louvain-la-Neuve, Belgium

*) corresponding author: simon.moser@empa.ch

1 Li calculation from discharge curves

$$c_{Li} = \frac{t \cdot I_{DC}}{V_{act} \cdot q}$$

where t is the lithiation time, I_{DC} is the discharge current, V_{act} is the volume of the absorber film, which can be occupied by Li, and q is the elementary charge.

Lithium is shown as the ratio with respect to the Cu concentration, c_{Cu}, which is calculated according to:

$$c_{Cu} = 1.75 \cdot \frac{\rho \cdot N_A}{M}$$

where ρ is the density of fully selenized CZTSe (5.7 g/cm^3)[1], N_A is the Avogadro constant and M is the molar mass of CZTSe. The pre-factor was chosen to reflect a Cu-poor stoichiometry properly.

2 Detailed ToF-SIMS data

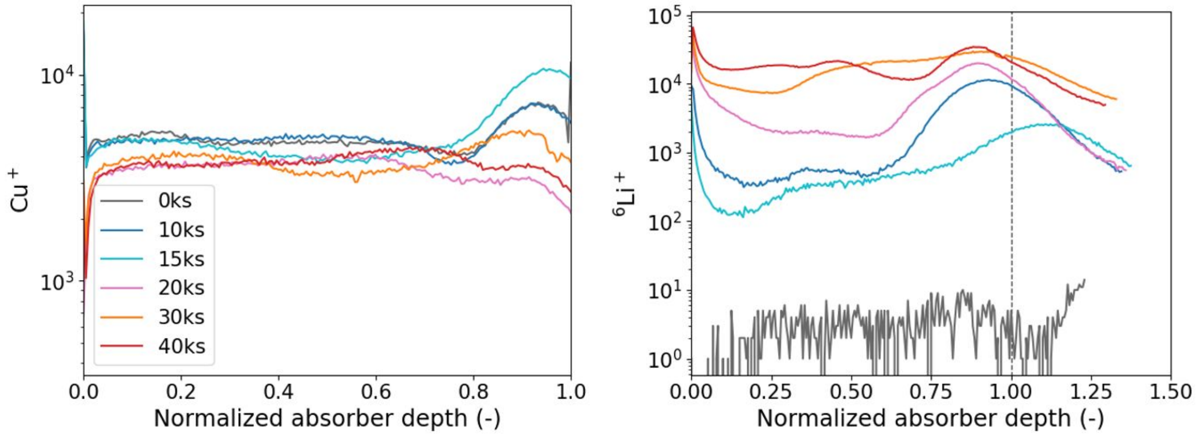

Figure S1: ToF-SIMS depth profile of Cu^+ and $^6Li^+$ of the various absorbers in the series.

3 XRD before and after annealing

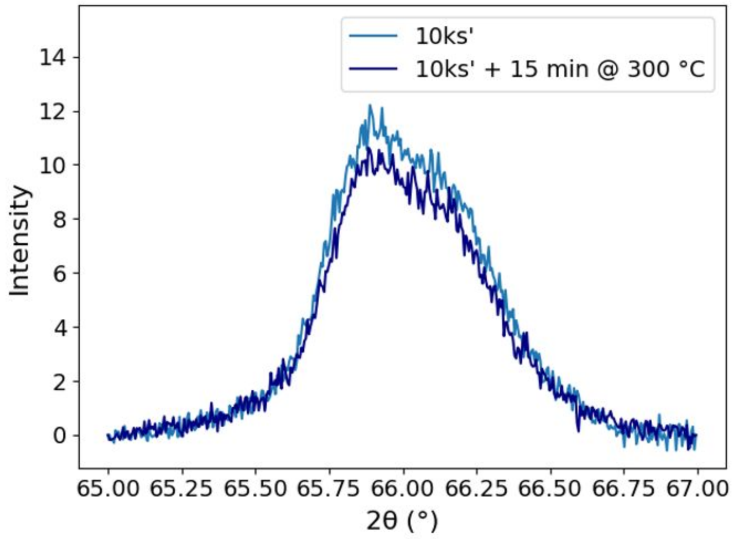

Figure S2: XRD zoom-in to 400 and 008 peaks before and after an additional heat treatment of 15 min at 300°C in N_2:Se protection atmosphere. The sample was fabricated identically as *10ks*.

4 Full-range XRD patterns

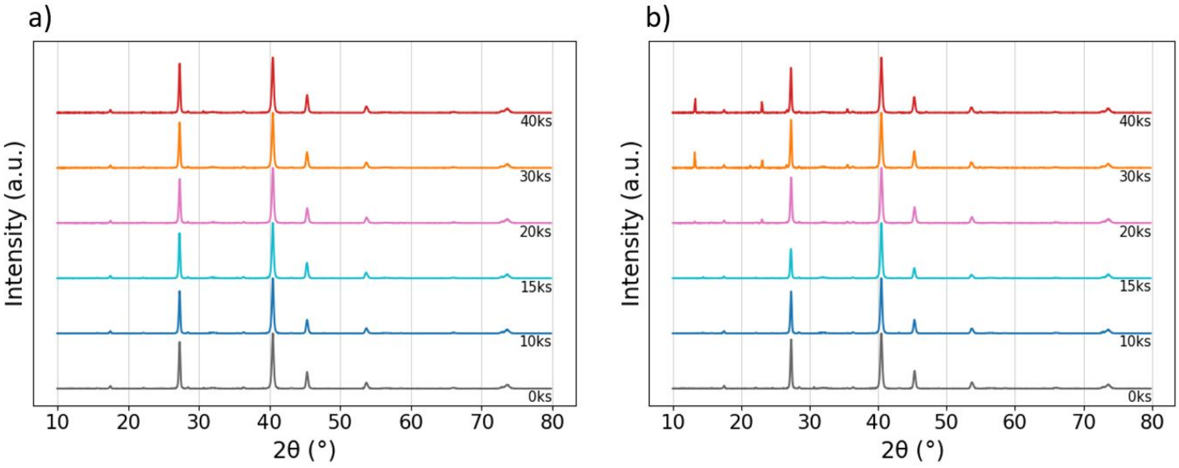

Figure S3: Full XRD diffraction patterns of a) pristine and b) electrochemically lithiated absorber.

5 ToF-SIMS and XRD analysis of adjacent spot

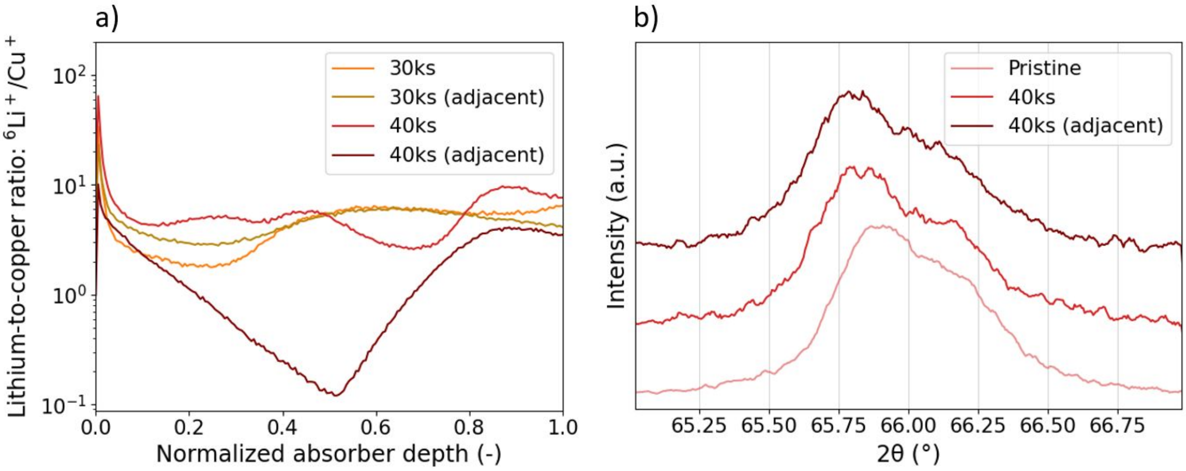

Figure S4: ToF-SIMS and XRD measurements of absorbers on and adjacent to the lithiation spot. a) The $^6Li^+/Cu^+$ ratio of *30ks* does not differ significantly between the lithiation spot and adjacent it. *40ks* shows a stronger discrepancy with the feature at ~0.5 absorber depth remaining unclear. b) XRD diffraction pattern zoom-in to the 400 and 008 reflexes of *40ks*. The measurement adjacent to the lithiation spot shows a comparable peak shift to the measurement on the lithiation spot.

6 Quantitative calibration

SIMS: $\frac{Li}{Li+Cu} \approx 0.013 \cdot {}^{Li}/_{Cu}$

XRD (112): $\frac{Li}{Li+Cu} \approx -16.7 \cdot \Delta d_{112}$

XRD (400): $\frac{Li}{Li+Cu} \approx -25.5 \cdot \Delta d_{400}$

Where *Li/Cu* is the ratio determined from ToF-SIMS, Δd_{112} is the difference in interplanar distance of the 112 plane and Δd_{400} is the difference in interplanar distance of the 400 plane.

7 XPS data

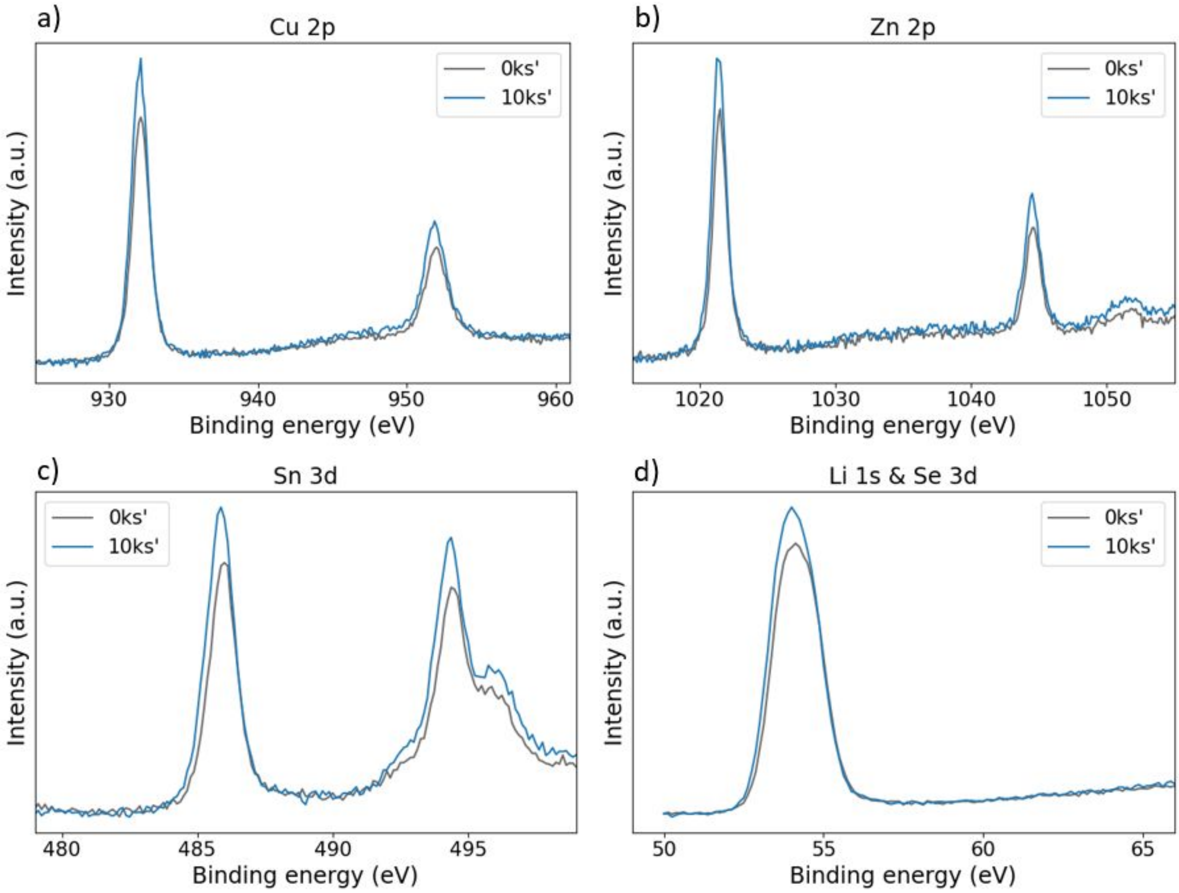
a)
Cu 2p
0ks'
10ks'
Intensity (a.u.)
930
940
950
960
Binding energy (eV)
b)
Zn 2p
0ks'
10ks'
Intensity (a.u.)
1020
1030
1040
1050
Binding energy (eV)
c)
Sn 3d
0ks'
10ks'
Intensity (a.u.)
480
485
490
495
Binding energy (eV)
d)
Li 1s & Se 3d
0ks'
10ks'
Intensity (a.u.)
50
55
60
65
Binding energy (eV)

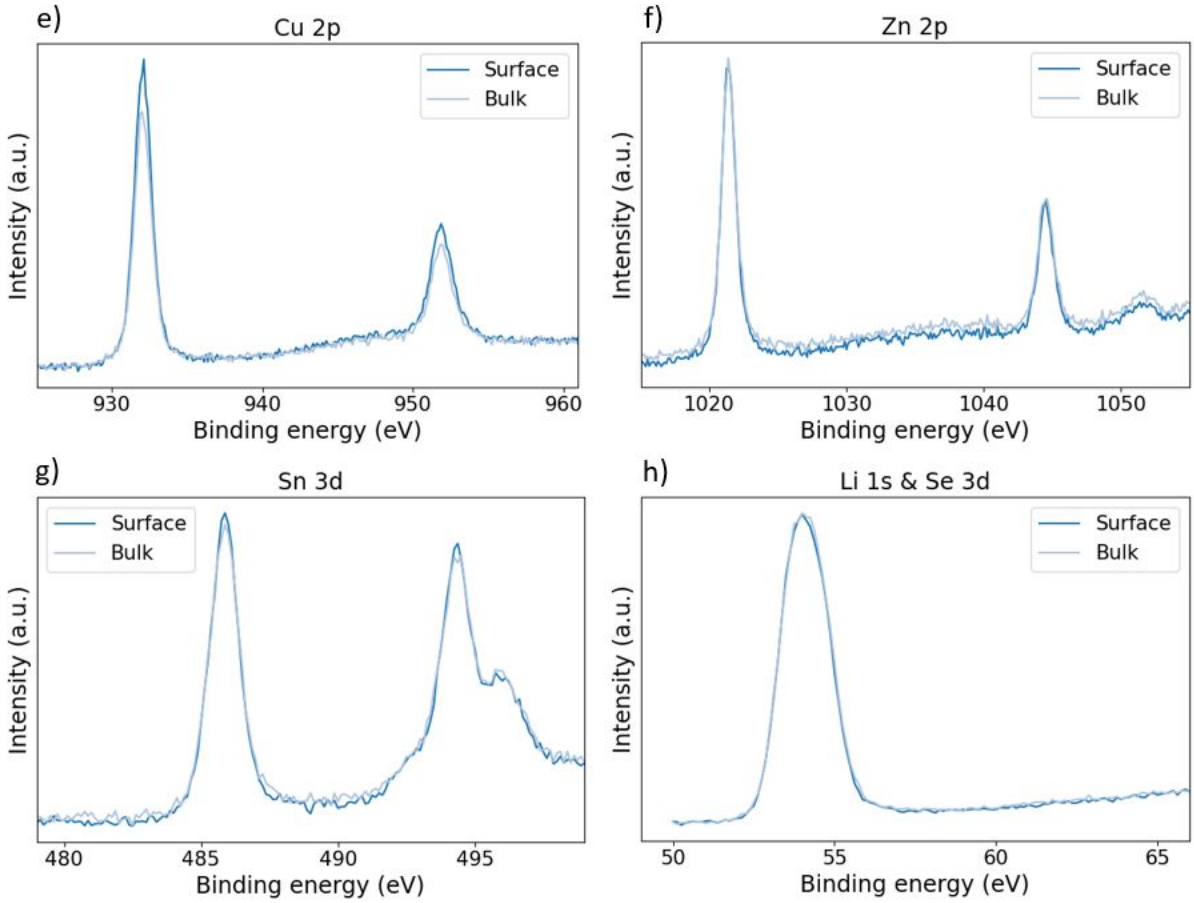

Figure S5: XPS data showing comparison between an untreated absorber *0ks*' similar to *0ks* and an absorber *10ks*' lithiated for 10'000 s Li similar to *10ks* in the proximity of the surface (a-d), revealing no significant differences upon electrochemical lithiation. XPS depth profiling compares a point in surface proximity and at roughly half of the absorber depth for *10ks* (e-h), revealing no significant gradients throughout the absorber depth. The Li 1s signal and the Se 3d signal are convoluted. The high abundance of Se in the material therefore prevents the accurate quantification or even detection of Li by XPS.

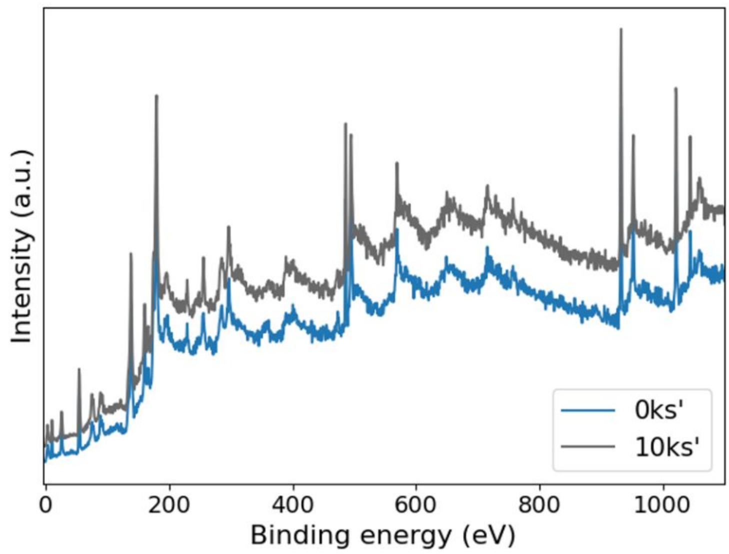

Figure S6: Full-range XPS spectra showing comparison between an untreated absorber *0ks'* similar to *0ks* and an absorber lithiated for 10'000 s Li similar to *10ks* in the proximity of the surface.

8 Discharge curves of all absorbers of the series

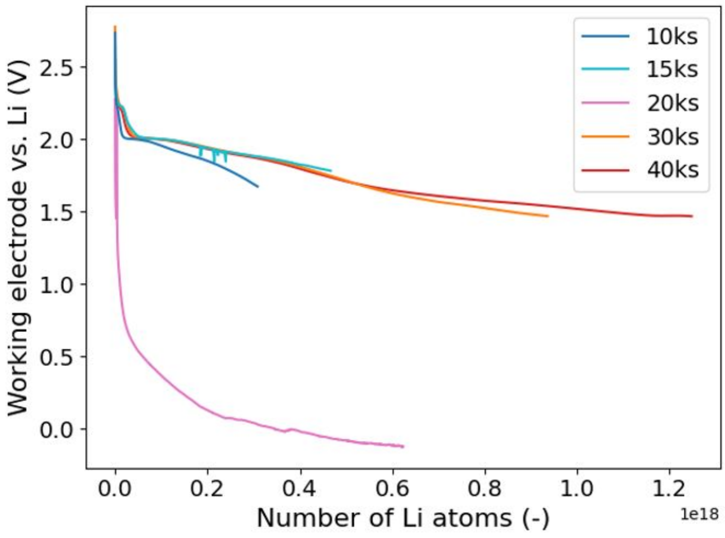

Figure S7: Discharge curve of *20ks* in comparison to the other discharge curves of the series.

9 PL spectra before and after lithiation

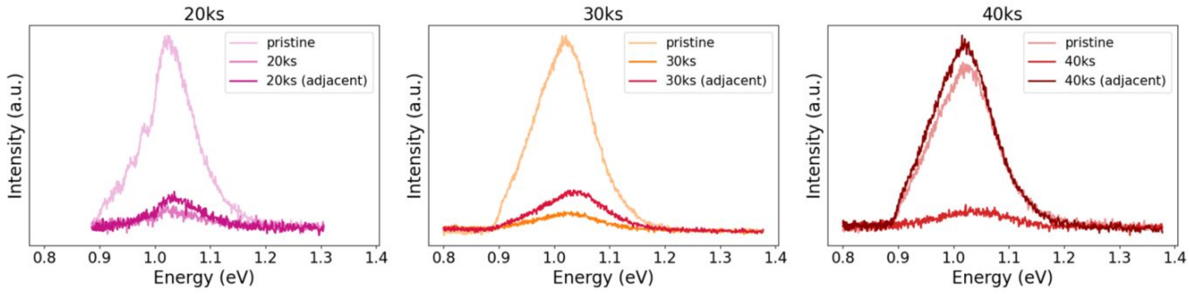

Figure S8: PL spectra of an absorber in its initial state and after 20'000 s electrochemical lithiation. The treated state was measured right on the spot, which was in contact with the electrolyte, and next to it.

10 Raman data

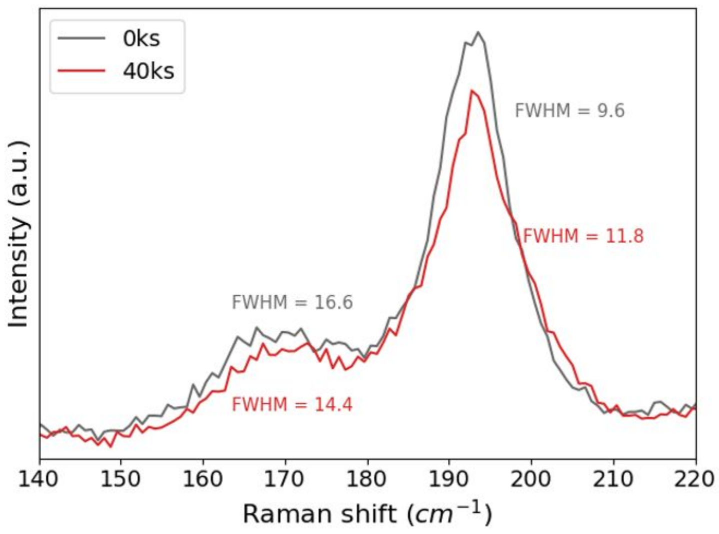

Figure S9: Raman spectra of an absorber in its initial state and after 40'000 s electrochemical lithiation (*40ks*). No general peak broadening is observed, ruling out the influence of a changed disorder state.

11 Additional treatment on different cell – JV properties

Table S1: JV properties of an additional cell, which experienced an identical treatment as the cell reported in the main manuscript.

	V_{OC} (mV)	J_{SC} (mA cm^{-2})	FF (%)	PCE (%)	$R_{s,\ ill.}$ (Ω cm^2)	$R_{p,\ dark}$ (Ω cm^2)
10'000 s Li repeated	489	32.0	55.6	8.7	2.1	20910

12 Additional characterization of 10'000 s Li device

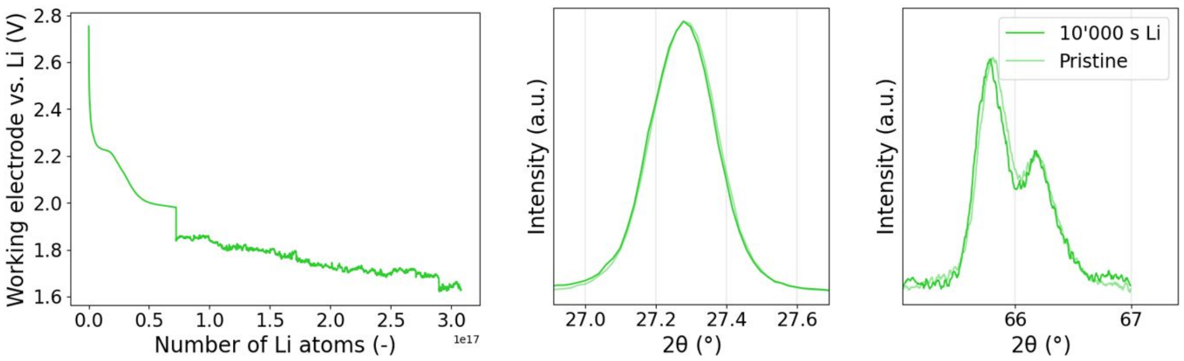

Figure S10: a) Discharge curve of 10'000 s Li device. b, c) 112 and 400/008 peak zoom-in obtained from XRD for 10'000 s Li device in pristine and lithiated state. Lattice parameter shift was found at 0.0019 Å and 0.0015 Å for *a* and *c*, respectively.

13 SEM micrographs

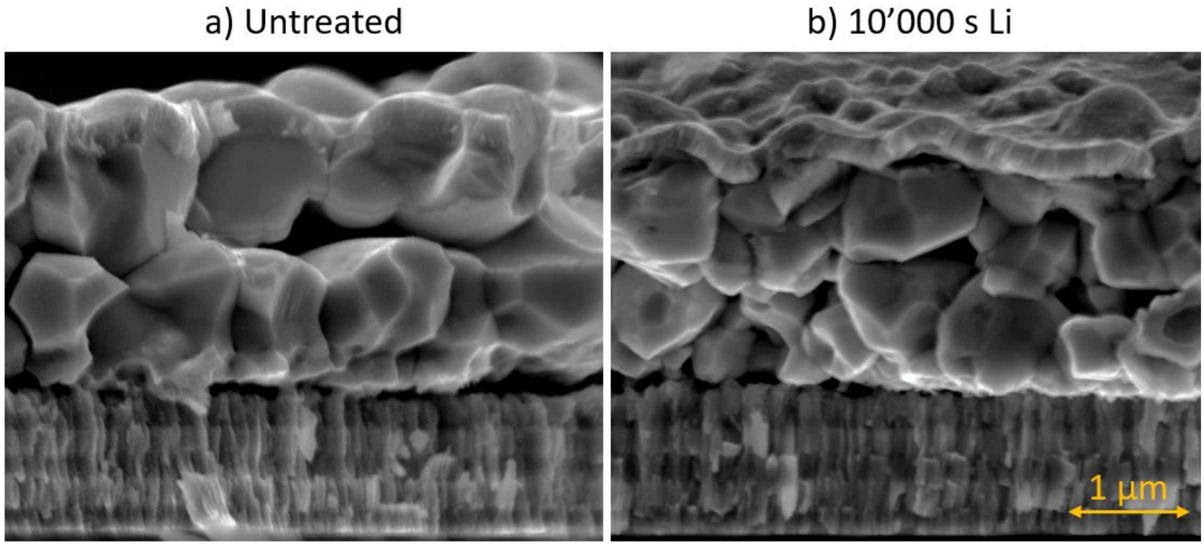

Figure S11: SEM cross-sectional images of initially Li-alloyed in (a) pristine state and (b) with an additional electrochemical treatment of 10'000 s.

14 V_{OC}-J_{SC} results

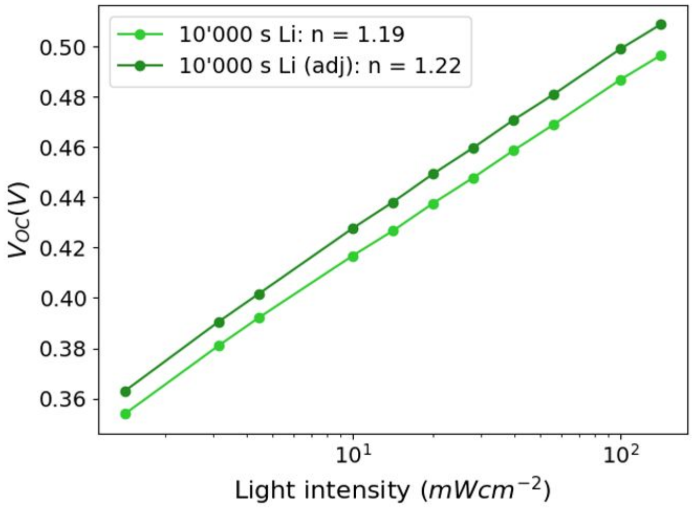

Figure S12: J_{SC}-V_{OC} measurements for the 10'000 s lithiated device with corresponding diode ideality factor on the lithiation spot and adjacent to it. Measurements were performed using a 100 W halogen lamp while varying the incident light using neutral density filters in the 1 – 142 mW cm^{-2} range.

15 T-dependent V_{OC} measurement

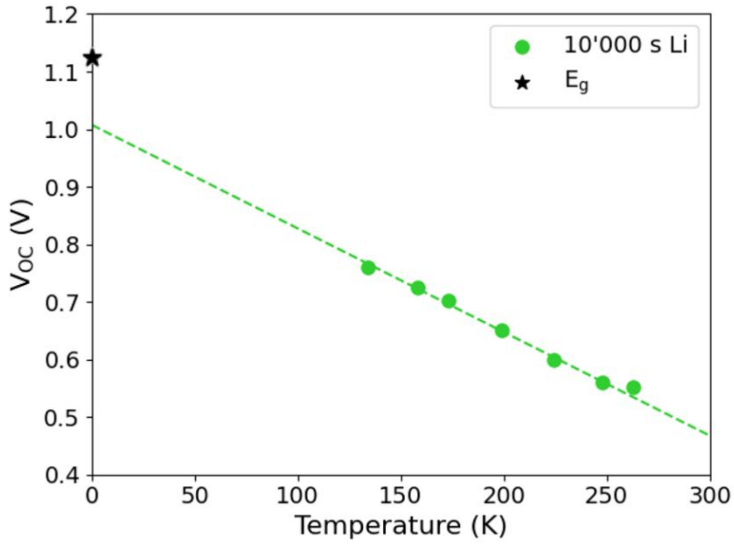

Figure S13: V_{OC}-T for the lithiated device. The band gap determined from EQE via the derivative method is marked with a black star.

16 Arrhenius plot C-f measurements

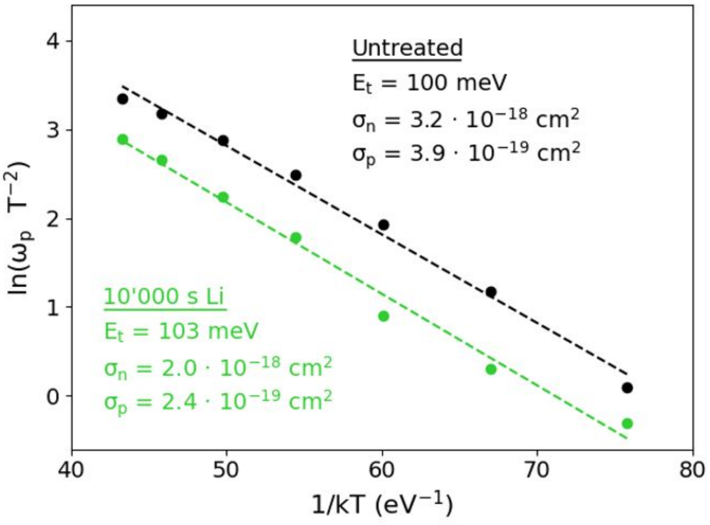

Figure S14: Arrhenius plot of capacitance-frequency measurements of an untreated and a lithiated device.

17 Additional results on adjacent cell

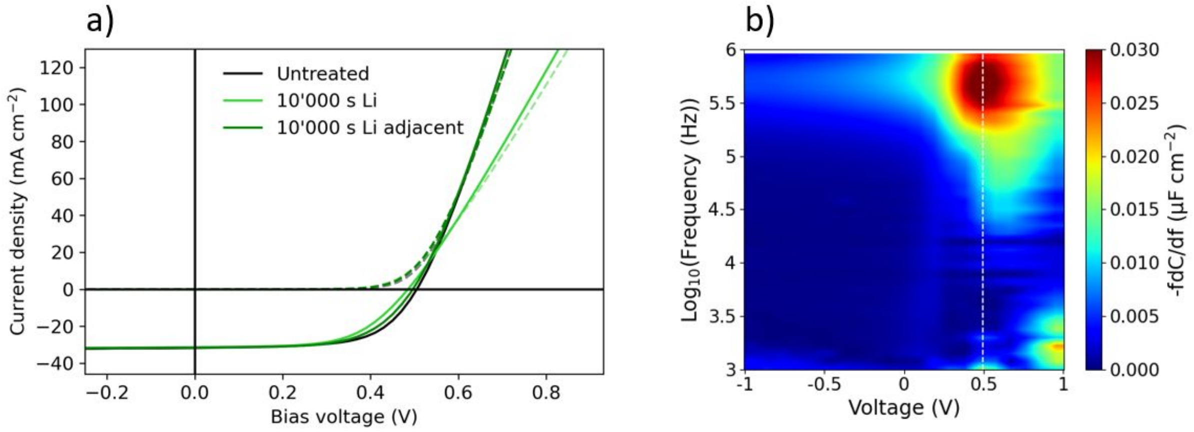

Figure S15: Additional characterization of the adjacent cell on lithiated sample, which was not in contact with the electrolyte. A) J-V curve of the adjacent cell shows a strong resemblance with untreated cell. There is no sign of deteriorated series resistance on adjacent cell. B) CVf "loss map" shows a less pronounced tail towards low voltages compared to Figure 6d.
